# Modus Operandi of a Pedalo-Type Molecular Switch: Insight from Dynamics and Theoretical Spectroscopy

**DOI:** 10.3390/molecules28020816

**Published:** 2023-01-13

**Authors:** Mario Taddei, Marco Garavelli, Saeed Amirjalayer, Irene Conti, Artur Nenov

**Affiliations:** 1 Dipartimento di Chimica Industriale, Università degli Studi di Bologna, 40136 Bologna, Italy; 2 Center for Nanotechnology, Center for Multiscale Theory and Computation, Physikalisches Institut, Westfälische Wilhelms-Universität Münster, 48149 Münster, Germany

**Keywords:** QM/MM, CASSCF/CASPT2, TD-DFT, mixed quantum-classical dynamics, time-resolved IR spectroscopy

## Abstract

Molecular switches which can be triggered by light to interconvert between two or more well-defined conformation differing in their chemical or physical properties are fundamental for the development of materials with on-demand functionalities. Recently, a novel molecular switch based on a the azodicarboxamide core has been reported. It exhibits a volume-conserving conformational change upon excitation, making it a promising candidate for embedding in confined environments. In order to rationally implement and efficiently utilize the azodicarboxamide molecular switch, detailed insight into the coordinates governing the excited-state dynamics is needed. Here, we report a detailed comparative picture of the molecular motion at the atomic level in the presence and absence of explicit solvent. Our hybrid quantum mechanics/molecular mechanics (QM/MM) excited state simulations reveal that, although the energy landscape is slightly modulated by the solvation, the light-induced motion is dominated by a bending-assisted *pedalo*-type motion independent of the solvation. To support the predicted mechanism, we simulate time-resolved IR spectroscopy from first principles, thereby resolving fingerprints of the light-induced switching process. Our calculated time-resolved data are in good agreement with previously reported measured spectra.

## 1. Introduction

**Figure 1:**
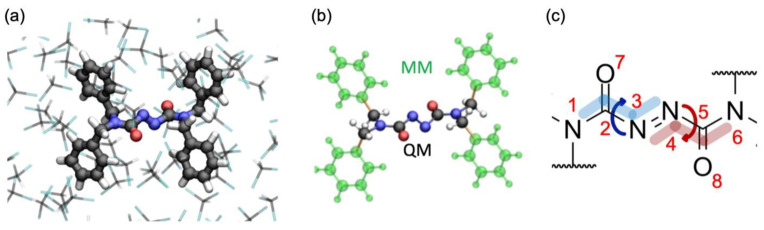
(**a**) QM/MM set up of the system (balls and sticks representation) in a chloroform solvent (stick representation). (**b**) Partitioning scheme of the solute; the QM region contains the azodicarboxamide unit, while the MM region contains the phenyl rings; bonds across which the QM/MM cut is applied are highlighted in orange. (**c**) Lewis structure of the photo-active azodicarboxamide unit with atom labeling; note that the GS geometry is not planar, showing a 75° distortion of the N_1_-C_2_-N_3_-N_4_ and N_3_-N_4_-C_5-_N_6_ dihedrals.

In recent decades, light-triggered molecular switches have been intensively studied not only because of their fundamental role in biological processes, such as vision [1], but also as promising candidates for the development of stimuli-responsive [2,3,4,5] functional materials, such as molecular devices [6,7,8,9], photo-responsive drug delivery systems [10], porous materials [11], liquid crystals [12] and optical memories [13], to name a few. Recently, a novel molecular photo-switch based on the azodicarboxamide chromophore unit (Figure 1) stepped into the spotlight thanks to a unique feature: an ultrafast photoinduced volume-conserving motion. Time-resolved UV-pump/IR-probe spectroscopy [14] supplemented with QM(TDDFT)/MM non-adiabatic molecular dynamics simulation on the solvated model [15] demonstrated that the chromophore, in contrast to other azo-compounds, does not undergo the usual *trans-cis* photoisomerization but rather a volume-conserving *pedalo*-type motion, rendering possible the application of this new class of chromophores under constrained conditions such as in solid state [16], on surfaces [17,18] and inside polymers [19].

The operation mechanism of the azodicarboxamide Is, however, a matter of debate. Based on microsolvation non-adiabatic molecular dynamics simulations at the CASSCF level, Liu and co-workers reported the usual *trans-cis* isomerization as the dominant decay mechanism [20]. Thus, a key factor for the exploitation of azodicarboxamide in different environments is to understand whether the *pedalo*-type motion is favored only in a medium which hinders the volume-demanding *trans-cis* process or whether it is an intrinsic property of the photo-switch. To elucidate the operation mechanism in detail and to evaluate in particular the coupling of the photochromic unit with the molecular environment, we report non-adiabatic molecular dynamics simulations on the isolated system and compare them with the dynamics on the solvated model. We find that, while the solvent affects the topology of the excited state (ES) potential energy surface (PES), the dynamical behavior is essentially unaltered. Moreover, to validate the finding and to allow a direct comparison with the experiments, we developed a protocol to simulate transient IR spectra based on the non-adiabatic molecular dynamics, which is applicable beyond the azodicarboxamide switching unit. Our results emphasize the importance of dynamical treatment of the photodynamic process.

## 2. Results and Discussion

### 2.1. Static Calculation: Critical Points and MEPs

**Figure 2:**
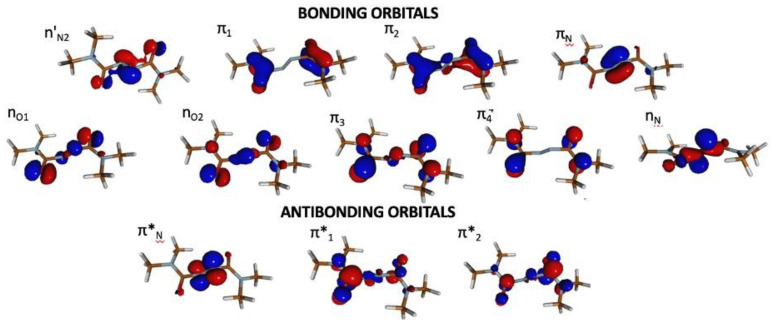
Active space (18,12) employed for CASPT2 calculations.

GS optimization in gas-phase at the DFT and MP2 level of theory show, in line with calculations on the solvated model [15], that the GS equilibrium of the photochromic unit is characterized by 75° distortion from planarity around the N_1_-C_2_-N_3_-N_4_ (α) and N_3_-N_4_-C_5_-N_6_ (β) dihedrals (Table 1, see Figure 1 for atom notation). It is worth noting that the two carbonyl groups are decoupled from the central C_2_-N_3_-N_4_-C_5_ (γ) fragment, i.e., the molecule does not exhibit a conjugated π-system in the GS. This is most clearly demonstrated by the frontier orbitals, $\pi_{N}$ and $\pi_{N}^{*}$, being essentially localized on the N=N fragment and decoupled from the remaining π-orbitals (see Figure 2).

The electronic structure analysis, carried out at the GS equilibrium geometry at the CASPT2 and TDDFT levels of theory (Table 2), shows that the lowest ES (S_1_), which appears in the visible range (below 3 eV), is characterized by a $n_{N}\;\to \;\pi_{N}^{*}$ transition involving orbitals localized on the N=N fragment (Figure 2) with a vanishingly small oscillator strength at equilibrium. A triad of ES appear near degenerate around 4.5 eV described by a $n_{O2}\;\to \;\pi_{N}^{*}$ transition (S_2_) and a pair of degenerate transitions from $\pi_{3}$ and $\pi_{4}$ to $\pi_{N}^{*}$ (S_3_ or S_4_). In passing, we note the different energy order of the aforementioned transitions at the CASPT2 and TD-DFT levels. In all three transitions, there is a net charge transfer from the carbonyl groups to the central fragment. Due to the decoupling of the carbonyl and N=N fragments, the oscillator strengths associated with these transitions are rather weak (Table 2). Overall, the electronic structure of isolated azodicarboxamide resembles closely that in chloroform [15].

**Figure 3:**
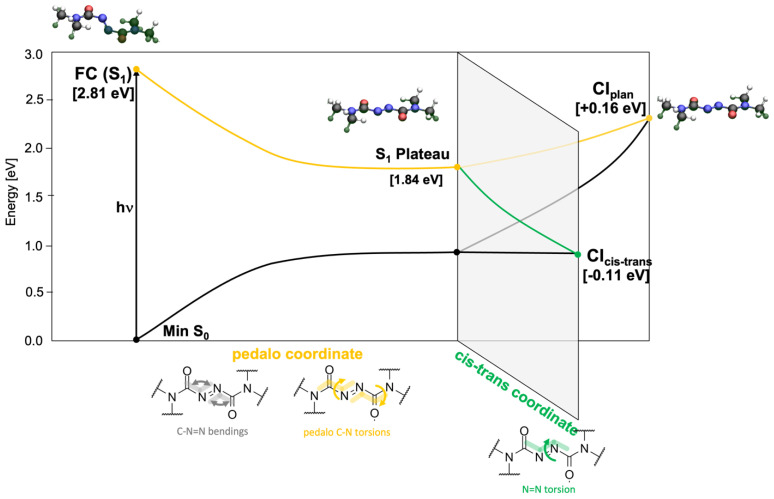
Scheme of the potential energy profile connecting the FC region of the S_1_ state to the S_1_/GS CI seam, obtained at QM(CASPT2)/MM level in gas phase based on relaxed scans along torsion and bending coordinates reported in Table S1 in the SI section.

Through analysis of the PES, we could trace the possible deactivation pathways which connect the FC region of the lowest electronic ES S_1_($n_{N}\pi_{N}^{*}$) to the CI seam with the GS (Figure 3). Optimization at both TDDFT/CAM-B3LYP and SS-CASPT2/SA-2-CASSCF(18,12) levels shows that the driving force for the vibrational relaxation from the FC point is the *pedalo*-type motion characterized by the simultaneous torsion around α and β, which leads to the planarization of the azodicarboxamide unit (both dihedrals acquire values close to 180°, Table 1), accompanied by the opening of the bending angles C_2_-N_3_-N_4_ (φ) and N_3_-N_4_-C_5_ (ϕ) from ca. 110° to 125°. Those modes drive the system toward a plateau on the PES of the S_1_ state (labeled S_1_ *Plateau* in Figure 3). The geometrical changes can be rationalized in the following way: in the closed shell GS the oxygen and nitrogen lone pairs (labeled $n_{O1}$, $n_{O2}$, ${n^{\prime }}_{N}$ and $n_{N}$) are all doubly occupied. Their spatial proximity would cause an increased steric repulsion in a planar azodicarboxamide, which would outweigh the gain from the conjugation in the π-system. Therefore, the chromophore adopts a highly twisted conformation. Upon S_1_ $n_{N}\;\to \;\pi_{N}^{*}$ excitation, the electron density in the nitrogen lone pair is depleted. The reduced steric repulsion facilitates the planarization thereby increasing the conjugation strength, evidenced by the constructive interference in the C_2_-N_3_ and N_4_-C_5_ segments of the anti-bonding orbital $\pi_{N}^{*}$ in the S_1_ *plateau* (see Figure S1 in the Supplementary Information, SI). To better accommodate the electron density in the plane, the angles φ and ϕ simultaneously widen to 125°.

We located two minimum energy CI along the S_1_($n_{N}\pi_{N}^{*}$)/S_0_ CI seam—labeled CI_cis-trans_ and CI_plan_—potentially relevant for the internal conversion to the GS, as shown schematically in Figure 3. While being almost isoenergetic, they are characterized by different structural parameters, documented in Table 1. CI_cis-trans_ exhibits torsion around the N-N bond (γ acquires a value of 112°) and lies about 0.1 eV below the S_1_ *Plateau*. In fact, it could be reached without an energetic barrier both at CAST2 and TDDFT level (green path in Figure 3). Conversely, CI_plan_ exhibits an overall planarization of the three dihedral angles, α, β and γ accompanied by a further widening of the angles φ and ϕ to ca. 135° (yellow path in Figure 3). CI_plan_ is located at about 0.15 eV above the S_1_ *Plateau.* We note the close resemblance of the two CIs with the planar and twisted S_1_($n_{N}\pi_{N}^{*}$)/S_0_ CIs found in azobenzene [21], which bears the same photoactive CNNC unit. The qualitative scheme of Figure 3 is based on relaxed scans along the *cis-trans* and *pedalo* coordinates carried out at the TDDFT and CASPT2 levels, documented in Figure S2 of the SI.

The S_1_($n_{N}\pi_{N}^{*}$) PES of the isolated azodicarboxamide resembles the one reported for the solvated model [15], exhibiting CIs with comparable geometrical features. Contrarily to what was reported in chloroform (CI_cis-trans_ and CI_plan_ lying ca. 0.2 eV above the S_1_ minimum), in gas-phase, no energy is present along the *trans-cis* coordinate. Thus, at least from an energetical point of view, this would suggest a higher propensity for the chromophore to undergo *trans-cis* isomerization in gas-phase instead of the *pedalo*-type motion.

### 2.2. Dynamics

Based on the static calculation we recognize two energetically competitive decay paths. Even though our previous dynamical study in chloroform demonstrated a clear preference for the *pedalo*-motion, it is not obvious the same dynamical behavior can be expected in gas-phase, in particular taking into account the stabilization of the CI associated with the *cis-trans* path. Static PES analysis has only a limited predictive power, as it neglects the role of the momentum conservation, which can divert the system from the minimum energy path. To obtain a more realistic picture of the mechanism, we run trajectory-based mixed quantum classical dynamics simulation.

**Figure 4:**
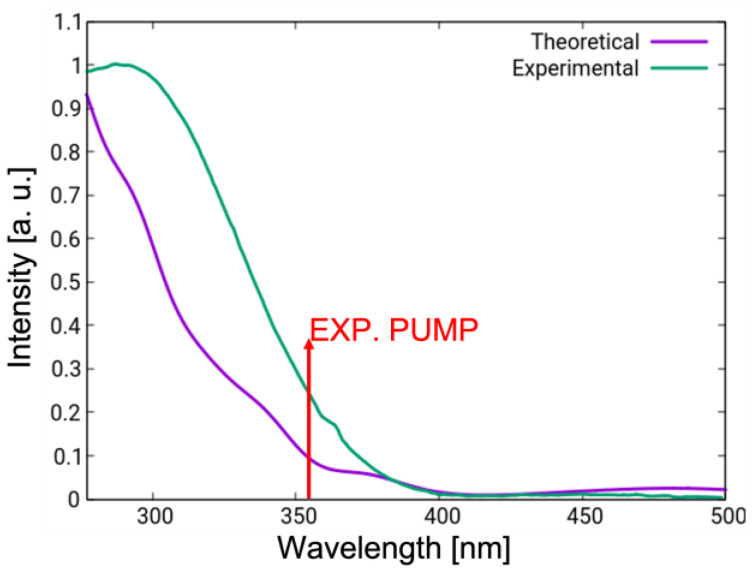
Linear absorption spectrum in chloroform simulated at the TD-DFT/CAM-B3LYP level on the basis of vertical excitation energies of 200 geometries obtained by means of Wigner sampling (violet) compared to the experimental spectrum (green). Red arrow indicates the central wavelength of the experimental pump pulse.

Figure 4 shows a comparison between the experimental (green) and simulated (violet) linear absorption spectra in chloroform. The UV-vis spectrum shows a weak band above 400 nm, which is attributed to the $n_{N}\;\to \;\pi_{N}^{*}$ transition (Table 2), and a more intense UV band due to the triad of states S_2_–S_4_. On the basis of this analysis, it can be deduced that the experimental pump pulse utilized to initiate the photoreaction in chloroform, centered at 355 nm (3.5 eV, indicated with a red arrow in Figure 4), addresses the manifold of states S_2_–S_4_. Mixed quantum classical dynamics reported previously in solvent were initiated in the brightest state of that manifold. Hence, the gas-phase simulations reported in this work follow the same selection criteria in order to allow a direct comparison.

**Figure 5:**
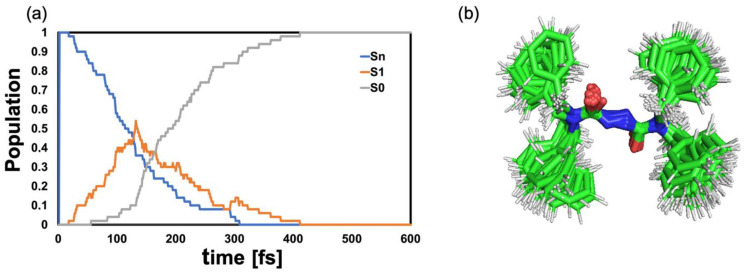
(**a**) Population dynamics in gas-phase. Sn comprises states S_2_–S_4_; the choice of the initial state is based on the oscillator strengths. (**b**) Superimposed geometries of the S_1_ → S_0_ hopping events for all trajectories.

Analysis of the statistics of the 50 trajectories shows that the internal conversion to the lowest electronic ES S_1_ occurs on a ~100 fs time scale. Repopulation of the GS is initiated shortly after and concluded within 400 fs (Figure 5a).

**Figure 6:**
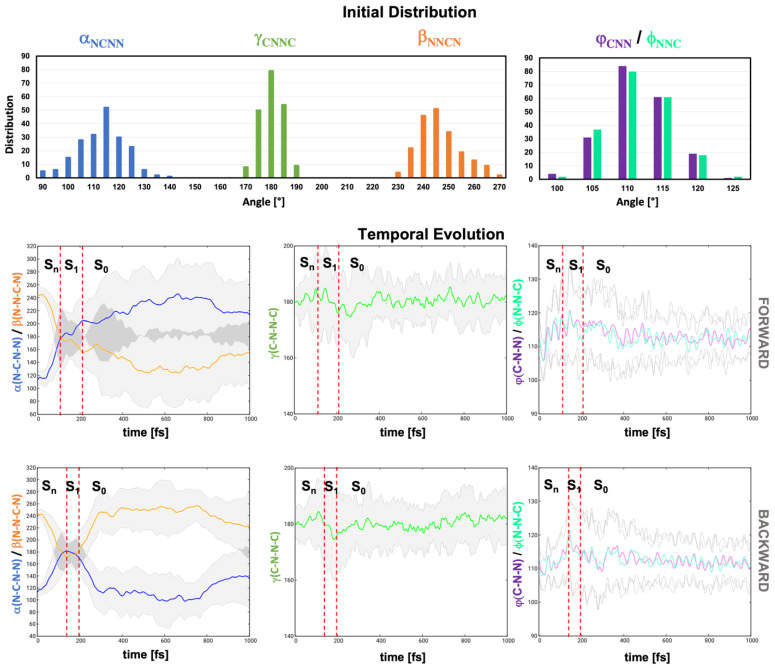
Initial distribution od dihedrals α (N_1_-C_2_-N_3_-N_4_) (blue), β (N_3_-N_4_-C_5--_N_6_) (orange), γ (C_2_-N_3_-N_4_-C_5_) (green) and angles φ (C_2_-N_3_-N_4_) (violet) and ϕ (N_3_-N_4_-C_5_) (top). Average evolution of α, β, γ, φ and ϕ undergoing forward (middle) and backward (bottom) *pedalo*-type motion. Gray areas represent the standard deviation with respect to the average value. Red dashed lines indicate average S_2_/S_1_ and S_1_/S_0_ hopping times.

To shed light into the geometrical deformations along the dynamics, we analyzed the variation of the torsional angles α and β, which act as markers for the *pedalo* mechanism, and of the torsional angle γ, representative of the *trans-cis* mechanism. The distribution of the values of those dihedrals in the equilibrium demonstrated the greater flexibility of α and β, peaking at 115° and 245°, respectively (i.e., ±65° with respect to the plane), with standard deviation of ca. 10°, with respect to γ, which barely deviates from planarity (Figure 6, top row). The rigidity γ is further evidenced by the analysis of the evolution of the average values of the three dihedrals. It demonstrates a propensity for the *pedalo*-type motion. While γ does not deviate from 180° along the dynamics, α/β exhibit a rapid concerted increase/decrease associated with the planarization of the azodicarboxamide unit on the time scale of 100 fs (Figure 6, middle and bottom rows). Moreover, planarization is initiated already in the manifold of the higher states S_2_–S_4_. Indeed, α and β reach average values of 168° and 187°, respectively, at the S_2_/S_1_ hopping geometries and approach 180° (α=177°, β=180°) at the S_1_/S_0_ hopping geometries (shown superimposed in Figure 5b). As anticipated in the previous section, the planarization is accompanied by alteration of the angles φ and ϕ. After a rapid decrease from their GS values of 112° to 107°, the average values of φ and ϕ progressively increase to 120° modulated by coherent oscillations with ca. 35 fs period (Figure 6, middle and bottom rows). After decay to S_0_, the angles return to the equilibrium values of 112°. The initial decrease is associated with the dynamics in the S_2_–S_4_ manifold, as confirmed by the average values at the S_2_/S_1_ hopping geometries (φ/ϕ=107°). Conversely, at the S_1_/S_0_ hopping geometries, φ and ϕ show values around 134°, as anticipated by the static analysis (Table 1). In fact, the φ/ϕ opening is necessary condition to reach CI_plan_, and at least one of the two angles φ and ϕ is found to have a value above 132° (see φ/ϕ distribution at the hopping geometries, Figure S3 in the SI).

The fact that the operation mechanism is dominated by the *pedalo*-type motion rather than the *trans-cis* isomerization, whose conical intersection CI_cis-trans_ is energetically more favored, can be rationalized by the conservation of momentum along the *pedalo*-type motion and the opening of φ and ϕ, activated upon decay to S_1_. By the time the φ/ϕ widening kicks in, torsion around α/β has already leads to the near planarization of the azodicarboxamide unit. As a consequence, the system reaches the S_1_/S_0_ CI seam, the torsional α/β modes are distributed around 180° ± 20°, which facilitated the forward *pedalo*-type motion upon crossing CI_plan_ and the completion the *pedalo*-cycle in the GS.

As noted earlier, the azodicarboxamide switch bears a certain resemblance to azobenzene. Azobenzene, whose lowest ES is of nπ∗ character, exhibits minimum energy S_1_/S_0_ Cis reminiscent of CI_plan_ and CI_cis-trans_. There is, however, a striking difference in their energetics. Whereas in azodicarboxamide, the two CIs are 0.2 eV apart and easily accessible out of the S_1_ *Plateau*, CI_plan_ is considerably destabilized on the S_1_ PES of azobenzene (ca. 0.8 eV above CI_cis-trans_). This makes it accessible only with sufficient kinetic energy at disposal. Indeed, in azobenzene, CI_plan_ acts as an efficient GS decay funnel only upon exciting the second ES (of ππ∗ nature). As in azodicarboxamide, it is the widening of the φ and ϕ angles, activated upon S_2_/S_1_ decay, which conserves enough momentum to drive azobenzene to CI_plan_ [21,22]. The relative stabilization of CI_plan_ in azodicarboxamide is a consequence of the smaller widening (i.e., smaller distortion) required to reach an S_1_/S_0_ degeneracy (φ/ϕ=135° compared to 145° in azobenzene). This is a consequence of the overall smaller S_1_–S_0_ energy gap in the S_1_ *Plateau* (0.95 eV compared to 1.85 eV in azobenzene) caused by the much stronger destabilization of the GS due to the increased in-plane electrostatic repulsion between the doubly occupied oxygen and nitrogen lone pairs. The increased accessibility of CI_plan_ makes the *pedalo*-type motion the exclusive decay mechanism in azodicarboxamide.

In conclusion, the presence, adjacent to the CNNC fragment, of a group bearing a lone pair (i.e., the carbonyl) increase the in-plane electrostatic repulsion and is responsible both for the non-planar GS conformation and for the dominance of the *pedalo*-type decay mechanism over the *trans-cis* isomerization. Consequently, the volume-conserving decay mechanism is an intrinsic feature of azodicarboxamide and is not enforced by the environment.

### 2.3. Spectroscopy

**Figure 7:**
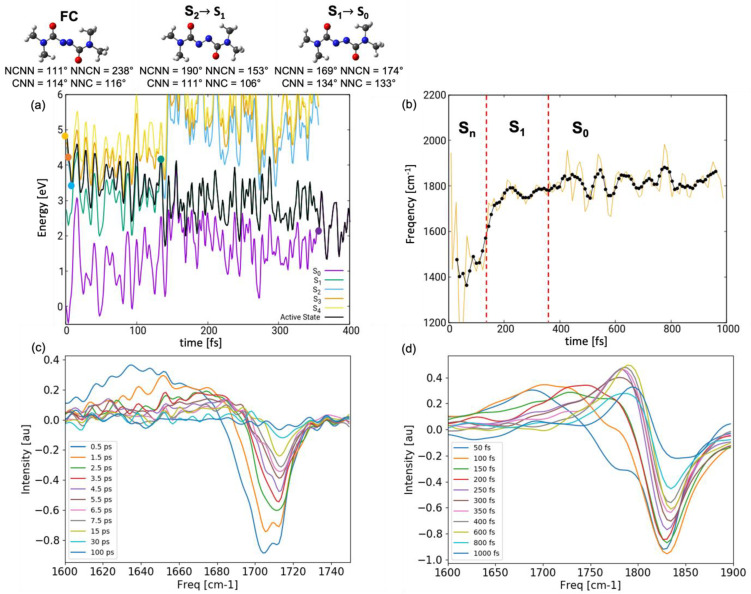
(**a**) Energetics of a representative trajectory together with conformational changes from the FC region toward CI_plan_: dynamics initiated in brightest state S_4_ (yellow), hopping events indicated by dots. (**b**) Time evolution of the frequency of the anti-symmetric carbonyl stretching along the representative trajectory show in (**a**); red dashed lines indicate the time of the S_2_/S_1_ and S_1_/S_0_ hopping events. (**c**) Experimental and (**d**) simulated UV-pump/IR-probe spectra in chloroform at various delay times.

To support atomistic interpretation, we simulated the transient IR spectra both in gas-phase and in chloroform (using the dynamics reported in our previous study [15]), following the procedure illustrated in the Methods section. In the following, we compare the simulated spectrum in chloroform with the experiment (the gas-phase spectrum is reported in Figures S4 and S5 in the SI) [14]. In particular, we focus on the carbonyl stretching absorbing in the spectral window 1600–1800 cm^−1^. Figure 7b shows the evolution of the instantaneous frequency $\omega \left(R\left(\tau \right)\right)$ along a representative trajectory shown in Figure 7a. It can be noted that immediately after excitation, when the system is in a higher lying ES (labeled as S_n_), the carbonyl stretching frequency red-shifts by 200 cm^−1^ to 1600 cm^−1^. A gradual blue-shift toward 1800 cm^−1^ is observed in the following 200 fs upon internal conversion before the trajectory returns to the GS after ca. 350 fs (indicated by a red vertical line in Figure 7b). Subsequently, the value of the carbonyl stretching continues to oscillate around 1800 cm^−1^. Vibrational analysis in the minima of the ground and excited electronic states at DFT/TDDFT level supports the observed trend. While in the GS, the symmetric and anti-symmetric stretching have a frequency of 1817 cm^−1^ and 1841 cm^−1^, in S_1_, we note red shifts to 1788 cm^−1^ and 1795 cm^−1^, respectively. Even more pronounced red shifts are observed in S_2_ with values of 1688 and 1753 cm^−1^. These red shifts can be understood by examining the frontier orbitals of the initially excited pair of degenerate electronic states S_2_ and S_3_, described through $\pi_{3}\;\to \;\pi_{N}^{*}$ and $\pi_{4}\;\to \;\pi_{N}^{*}$ transitions, respectively: (a) $\pi_{3}$ and $\pi_{4}$ show bonding contribution on the carbonyl fragment (Figure 2), which is weakened upon exciting an electron; (b) upon planarization, the carbonyl and diazo fragment align and the $\pi_{N}^{*}$ orbital delocalizes over the azodicarboxamide unit, thereby exhibiting anti-bonding contribution on the carbonyl fragment. Thus, upon excitation and planarization, the C=O bond is weakened which is resembles in the pronounced red shift of the associated stretching modes. A more modest red shift is observed in S_1_($n_{N}\pi_{N}^{*}$) as $n_{N}$ has no contribution on the carbonyl group and the hole created therein upon excitation does not weaken the C=O bond.

The transient UV-pump/IR-probe spectrum resolves the red shift of the carbonyl stretching mode in the ES as a positive signal peaking at 1700 cm^−1^ at early times (50 fs in Figure 7d) and progressively blue-shifting toward 1780 cm^−1^ in the next 300 fs. From there on, the red (blue) shift is slowed down but is clearly visible in the diminishing intensity of the overall signal indicating a destructive interference with the bleach around 1825 cm^−1^ present in the spectra due to the depletion of the GS. The persisting res-shift after the return to the GS is due to the cooling of the vibrationally hot wave packet, a process which takes several picoseconds and is, thus, not concluded within the simulation window.

The simulated spectrum reproduces qualitatively the experimental one (Figure 7c), though we note several quantitative discrepancies. The simulated spectrum is blue-shifted (e.g., the bleach has a 100 cm^−1^ blue-shift). This is due to a well-known tendency of DFT to overestimate normal mode frequencies. In fact, functional-specific scaling factors have been derived for GS frequency calculations aimed at correcting this error. As no scaling factors are available for electronic ES, we chose not to apply such a correction protocol. More striking is the difference in the time scales of the simulated and experimentally observed dynamics. The simulations predict a rapid blue-shift within 0.4 ps associated with ultrafast decay to the GS through CI_plan_. Thus, one could argue that the entire ES dynamics may remain concealed to the experiment, with the first measurement reported only after 0.5 ps. Here, we argue that the disagreement is a consequence of the strong overestimation of the decay rate in the simulations. First, the simplified hopping routine relying on the diminishing energy gap equates the hopping probability to unity every time the two surfaces come close. In reality, hopping will be promoted via vibronic coupling along selected modes, thus slowing down the decay. Second, the energy at which the dynamics is initiated in S_2_–S_4_ (both in gas-phase and in solution) is ca. 1 eV higher compared to the wavelength (355 nm/3.50 eV) at which the system is pumped experimentally (indicate with a red arrow in Figure 4). Upon internal conversion to S_1_, this energy in converted into kinetic energy, which facilitates the approach to the S_1_/GS CI region and accelerates the decay. This hypothesis is supported by the outcome of a trajectory initiated without initial kinetic energy in the S_1_ state (comparable to pumping at 500 nm/2.50 eV, Figure S4 in the SI). We observe an increase of ES lifetime by almost an order of magnitude both in gas-phase (hop to the GS after 679 fs) and in solvent (hop after 888 fs). We note that in both cases, the system follows the bending-assisted *pedalo*-type mechanism, i.e., the few hundred femtoseconds spent roaming in the S_1_ *Plateau* are not enough to activate modes toward the energetically more favored CI_cis-trans_.

In light of this arguments, we can be quite confident that at delay time Δt=500 fs, the experiment resolves the later dynamics in S_1_ missing out the motion occurring in the initially excited higher-lying states. This explains why the excitation-induced red shift in the simulated spectrum (>100 cm^−1^ difference between maximum of the positive and negative peak at Δt=50 fs) is more pronounced compared to the experiments (ca. 50 cm^−1^ difference between maximum of the positive and negative peak at Δt=500 fs).

Overall, the resemblance between the theoretical and experimental transient IR spectra validates the molecular dynamics protocol a strongly supports the dominance of the *pedalo*-type mechanism over *trans-cis* isomerization.

## 3. Methods

All the calculations reported in this work were performed with the COBRAMM [23] QM/MM interface, which links the QM packages Gaussian09 [24] and OpenMolcas [25], used to describe the photo-responsive core of the system with the MM suite AMBER [26], used for describing the environment.

### 3.1. QM/MM Setup

For the solvated system, a representative geometry embedded in a droplet of chloroform with radius 15 Å was obtained by sampling the conformational freedom by means of a classical MM simulation as described in Ref. [15]. A High-Medium-Low layer partitioning was employed in which the central photoactive moiety of the chromophore is described at QM level (High layer), while the four phenyls substituents, as well as chloroform, are treated at the MM level (Figure 1a). In the MM calculation, the azodicarboxamide is parametrized using the GAFF [27] force field [15], while chloroform is described following the work of Cieplak et al. [28]. The High layer-Medium layer cut was placed between the sp^3^- and sp^2^-hybridized carbons of the linker group and of the phenyl ring, respectively, as indicated in Figure 1b. The phenyl rings and solvent molecules within 9 Å of the solute were allowed to move (Medium layer), while the remaining solvent molecules were kept fixed. Concerning the gas-phase model, the initial setup has been obtained by stripping the solvent and re-optimizing the chromophore employing a High-Medium layer partitioning.

### 3.2. Electronic Structure Calculation

For both the gas-phase and solvated model, the basis set employed is 6-31G*. Electronic ground state (GS) calculations were performed at the density functional theory (DFT) level using the functional CAM-B3LYP [29] and at the Møller Plesset second-order perturbation theory (MP2) [30] level. ES energies in the Frank–Condon region were calculated using a time-dependent (TD) version of DFT, again using the functional CAM-B3LYP, and with complete active state self-consistent field (CASSCF), averaging over seven states, corrected by second–order multi-reference perturbation theory (CASPT2) in its single state (SS) flavor [31]. The active space includes the valence π-orbitals and the oxygen and nitrogen lone-pairs of the chromophore, leading to a total of 18 electrons in 12 orbitals (Figure 2). We will refer to the level of theory as SS-CASPT2/SA-7-CASSCF (18,12). Critical geometries along the lowest ES were optimized at the same computational level; however, only the lowest two state were used in the state-averaging to reduce the computational cost. Conical intersection optimizations were performed at the QM(CASPT2)/MM level with the gradient projection algorithm of Bearpark et al. [31,32] implemented in COBRAMM.

### 3.3. Linear Absorption Spectra in Chloroform

In total, 200 geometries were generated around the GS equilibrium using the Wigner sampling technique through an interface with a stand-alone script, part of the quantum molecular dynamics program JADE [32]. The sampling was performed at room temperature (300 K) and high-frequency modes belonging to C-H and N-H vibrations were excluded from the sampling. Each of the sampled geometries was subjected to a 10-ps long MM equilibration run, in which the QM region was kept fixed through the harmonic constraints, allowing the MM region to adapt at each sampled geometry. The linear absorption spectrum was simulated according to Equation (1):

(1) $$I\left(\omega \right)\propto \underset{n}{\sum }\underset{e}{\sum }f_{e\leftarrow g}\left(R_{n}\right)e^{\left(-\frac{\Delta E_{ge}{\left(R_{n}\right)}^{2}}{2\sigma^{2}}\right)}$$

where $f_{e\leftarrow g}\left(R_{n}\right)$ and $\Delta E_{ge}\left(R_{n}\right)$ denote the geometry dependent oscillator strength and excitation energy at given geometry coordinates $R_{n}$ and σ is the standard deviation used to account phenomenologically for the spectral line shape. The sums run over the number of snapshots (*n*) and over the manifold of excited states I. The single point calculations were performed at the QM(TD-DFT)/MM level of theory using a uniform value for σ=0.1 eV.

### 3.4. Mixed Quantum Classical Dynamics Gas-Phase

Mixed quantum-classical dynamics simulations in gas-phase were performed at the QM(TD-DFT)/MM level of theory considering four ES. In total, 50 initial conditions were generated using the protocol outlined in Section 3.3. Thereby, the initial velocities were obtained by combining the velocities of the QM part (from the Wigner sampling) with those of the MM atoms (the four phenyl substituents) from the last snapshot of the MM equilibration. Each trajectory was initiated in the brightest of the triad state below 400 nm (labeled S_2_–S_4_, see the discussion of the electronic structure in Section 2.1. for details) and propagated for 2 ps using a time step of 1 fs. Nonadiabatic events were evaluated with a simplified scheme relying on the energy gap as a criterium for changing the electronic state with a threshold of 2 kcal/mol. Back hopping was always allowed between excited states, while it was forbidden once the trajectory hopped to the electronic GS.

### 3.5. Theory and Implementation of the Time-Resolved IR Spectroscopy Simulations

We have simulated the transient UV-pump/IR-probe spectra both in gas-phase and in solvent. To this aim, we made use of the procedure employed by Stock et al. [33]. Working in the weak regime in which light–matter interactions can be treated perturbatively, we consider a system described by the Hamiltonian $\hat{H}$, which at time *t* = 0 is excited by an ultrashort optical pump pulse to an electronic ES. In the dipole approximation, the time-dependent interaction with the electric field is described by a time-dependent operator ${\hat{H}}_{int}={\hat{\mu }}^{OPT}E\left(t\right)$, where ${\hat{\mu }}^{OPT}$ is the transition dipole moment operator and $E\left(t\right)$ is the electric field. The excitation is assumed to be impulsive, implying that the nuclear wave packet, which before the arrival of the pump pulse is at equilibrium in the GS, is projected vertically on the ES where it starts to evolve. The field-free evolution is governed by the Schrödinger equation (Equation (2)):

(2) $$\Psi \left(\Delta t\right)=e^{-\frac{i}{\hbar }\hat{H}\Delta t}\Psi \left(0\right)$$

While the exact solution can be obtained by means of explicit quantum dynamics, the Schrödinger equation can be solved with some approximations by means of semi-classical and mixed quantum-classical approaches. At time Δt after the interaction with the pump pulse, the system interacts with an IR probe pulse, which promotes it to a higher vibrational level on the same electronic state. The interaction with the pulse perturbs the system creating an oscillating electric dipole which generates a macroscopic polarization $P\left(\Delta t,\tau \right)$, itself the source of an electric field emitted by the system during the time interval τ after the interaction with the probe pulse [34]. Mathematically, the polarization is described by the following equation (Equation (3)):

(3) $$P\left(\Delta t,\tau \right)=\frac{i}{\hbar }\Psi \left(0\right)|{\hat{\mu }}^{OPT}e^{+\frac{i}{\hbar }\hat{H}\Delta t}e^{+\frac{i}{\hbar }\hat{H}\tau }{\hat{\mu }}^{IR}e^{-\frac{i}{\hbar }\hat{H}\tau }{\hat{\mu }}^{IR}e^{-\frac{i}{\hbar }\hat{H}\Delta t}{\hat{\mu }}^{OPT}|\Psi \left(0\right)$$

Which is the expectation value of the dipole operator. $\Psi \left(0\right)$ denotes the system, initially in its electronic GS. Following the interaction with the pump pulse (formally two wave-matter interactions, one with the *bra* and one with the *ket*), the system evolves on the ES surface during the time interval Δt. Subsequently, an interaction with an IR pulse brings the (*ket*) wave packet to a vibrational ES. During the time interval τ after the IR pulse, the left- and right-hand side are propagated under the influence of a different vibrational Hamiltonian, a phenomenon termed coherence. The electronic density oscillating between two quantized vibrational levels during the time τ is the source of an emitted field with a frequency proportional to the energy difference between the vibrational levels. Although the interaction of the pump and probe pulses with the system induces a third-order nonlinear response, replacing the zero-order stationary initial state $\Psi \left(0\right)$ by a first-order nonstationary (excited) state $\Psi^{*}\left(\Delta t\right)$, which is time-dependent due to the initial preparation of the system, it is possible to describe the polarization by following linear expression (Equation (4)) [35]:

(4) $$P\left(\Delta t,\tau \right)=\frac{i}{\hbar }\Psi^{*}\left(\Delta t\right)|e^{+\frac{i}{\hbar }\hat{H}\tau }\hat{\mu }e^{-\frac{i}{\hbar }\hat{H}\tau }\hat{\mu }|\Psi^{*}\left(\Delta t\right)$$

In the semi-classical line shape theory [36,37], the coherence dynamics can be approximated by the fluctuation of the energy gap $\omega \left(\tau \right)$ between the two vibrational levels along the trajectory (Equation (5)):

(5) $$P\left(\Delta t,\tau \right)=\frac{i}{\hbar }\Psi^{*}\left(\Delta t\right)|\hat{\mu }e^{-\frac{i}{\hbar }\overset{\Delta t+\tau }{\underset{\Delta t}{\int }}\omega \left(R\left(t^{\prime }\right)\right)dt^{\prime }}\hat{\mu }|\Psi^{*}\left(\Delta t\right)$$

The time dependence of the energy gap is related to the evolution of the coordinates $\omega \left(\tau \right)=\omega \left(R\left(\tau \right)\right)$. In the harmonic approximation, the energy difference between two vibrational levels is ℏω, thus equal to the frequency of the vibrational mode.

In practice, the trajectory is propagated during the interval τ subject to the ES Hamiltonian as if there was no interaction with the IR pulse, and at every time step, the instantaneous frequency of the mode(s) of interest is calculated.

**Figure 8:**
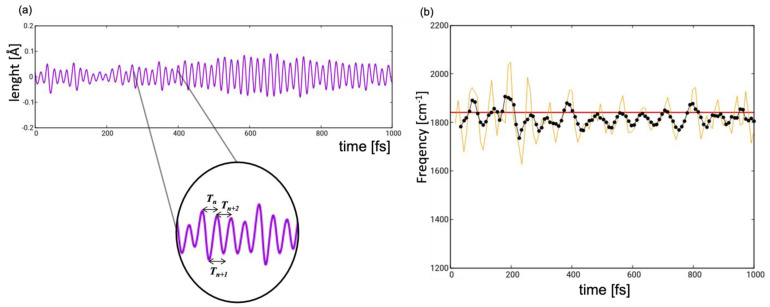
(**a**) Oscillating function representing the antisymmetric stretching which originates from subtracting the values of the two carbonyl groups at each time step along a representative trajectory. (**b**) Time evolution (yellow line) of the frequency of the antisymmetric stretching mode computed as the inverse of the time interval between two consecutive maxima or minima (see inset of (**a**)); black dotted line indicates the time-evolution of frequency averaged over five periods; red horizontal line represents the frequency of the antisymmetric stretching calculated at the GS equilibrium structure at DFT/CAM-B3LYP level.

The experimental spectrum was recorded in the frequency range associated with the carbonyl stretching [14]. For both types of light-induced mechanisms—*pedalo*-type motion and *trans-cis* isomerization—the C=O groups are not directly involved but rather act as a spectator mode of the nuclear dynamics, whose frequencies are modulated in the course of the internal conversion in response to the changing electronic structure. To obtain the instantaneous frequency $\omega \left(R\left(\tau \right)\right)$, we tracked the change of the value of the anti-symmetric (we note that in transient IR spectroscopy only anti-symmetric modes are active) carbonyl stretch along each trajectory defined as $\left[CO_{1}\left(R\left(\tau \right)\right)\;-\;CO_{2}\left(R\left(\tau \right)\right)\right]\;÷\;2$, where $CO_{1}\left(R\left(\tau \right)\right)$ and $CO_{2}\left(R\left(\tau \right)\right)$ are the value of the two CO distances at each time instance $\tau =\Delta t+t^{\prime }$. Figure 8a shows the evolution of the anti-symmetric carbonyl stretching value along a representative trajectory. We observe a periodically oscillating function with a period of ca. 20 fs. The instantaneous frequency was then obtained as the inverse of the time interval between two consecutive maxima or minima, as shown in the inset of Figure 8a. The data processing results in a dataset of instantaneous frequencies in ca. 10 fs intervals (yellow profile in Figure 8b). To smoothen the profile and minimize the effects of outliers, we averaged over five oscillation periods $\omega \left(R\left(\tau_{i}\right)\right)=\frac{1}{5}\sum_{k=i-2}^{i+2}\omega \left(R\left(\tau_{k}\right)\right)$.

In mixed quantum-classical theory, the wave packet is approximated by a swarm of trajectories each one propagated independently, and the total polarization is computed by summing over all trajectories (Equation (6)):

(6) $$P\left(\Delta t,\tau \right)=\frac{i}{\hbar }\frac{\mu^{2}}{N}\overset{N}{\underset{n}{\sum }}e^{-\frac{i}{\hbar }\overset{\Delta t+\tau }{\underset{\Delta t}{\int }}\omega \left(R_{n}\left(t^{\prime }\right)\right)dt^{\prime }}$$

where n runs over the total of N trajectories and μ is the magnitude of the IR intensity assumed to be coordinate independent. Fourier transformation of the polarization with respect to the interval τ gives the transient IR spectrum (Equation (7)):

(7) $$I\left(\Delta t,\omega \right)=2Re\overset{\infty }{\underset{0}{\int }}P\left(\Delta t,\tau \right)e^{-i\omega \tau }e^{-\tau /2T_{1}\;}d\tau$$

where we introduce a damping function $e^{-\tau /2T_{1}\;}$, with $T_{1}$ as the phenomenological lifetime of the coherence due to the dephasing of the signal. In this work, $T_{1}$ was set to 300 fs.

Finally, the transient difference spectrum is calculated as (Equation (8)):

(8) $$\delta I\left(\Delta t,\omega \right)=I\left(\Delta t,\omega \right)-I_{0}\left(\omega \right)$$

where the reference spectrum $I_{0}\left(\omega \right)$ represents the steady spectrum of the GS. In practice, $I_{0}\left(\omega \right)$ is obtained with the same protocol as $I\left(\Delta t,\omega \right)$ by running the same set of 50 trajectories in the GS for the duration of 2 ps.

We are aware that there are more accurate protocols to obtain the instantaneous frequencies $\omega \left(R\left(\tau \right)\right)$, a notable example being the mode tracking procedure by Neugebauer et al. [38]. Our protocol is justified by the observation that the carbonyl stretching couples only weakly to other modes. To support these statements, the IR spectrum in the GS $I_{0}\left(\omega \right)$ gives a steady spectrum peaking at 1825 cm^−1^, in good agreement with the analytically computed value for the anti-symmetric stretching of 1841 cm^−1^.

## 4. Conclusions

The recently reported peculiar behavior of azodicarboxamide to undergo a volume-conserving *pedalo*-type decay mechanism upon excitation has attracted attention due to the potential exploration as a molecular switch in confined environments [14,15]. In contrast to chemically related dyes containing a central diazo unit, such as azobenzene, azodicarboxamide does not undergo volume-demanding *cis-trans* isomerization. In the present contribution, by means of mixed quantum classical dynamics simulation at the QM/TD-DFT)/MM level, we demonstrate that the *pedalo*-type motion is an intrinsic feature of the system determined by its electronic composition, in particular by the presence of carbonyl groups adjacent to the diazo unit. The strong electrostatic repulsion between the electron densities of the spatially proximate oxygen and nitrogen lone pairs to which a planar azodicarboxamide would be exposed to outweigh the potential gain from the conjugation. This forces a 75° twist along the NCNN/NNCN dihedrals in the *trans* isomer in the electronic GS, contrarily to azobenzene dyes, which are planar in their *trans* isomer. Instead, the central CNNC fragment is rather stiff and does not deviate from 180°. Photoexcitation with visible to mid-UV light excites a higher lying excited state (S_2_–S_4_), depleting the electron density in the lone pairs. This creates a pronounced drive toward planarization (and an increase of the conjugation strength). Notably, a near complete planarization is observed within the first 100 fs, i.e., even before the system decays to S_1_. The electronic structure S_1_ ($n_{N}\pi^{*}$) resembles that of the lowest electronic state of azobenzene and, in accordance, induces a widening of the CNN/NNC bending angles which ultimately steers the system towards a region on the S_1_/S_0_ CI seam, where the central dihedral CNNC remains planar, thus denoted CI_plan_. In contrast to azobenzene, where reaching CI_plan_ requires pronounced CNN/NNC bending deformation (ca. 145°) and, thus, a considerable amount of activation energy (ca. 0.7 eV), in azodicarboxamide, the CI_plan_ can be accessed via minor bending deformation (ca. 135°) and, correspondingly, a smaller barrier (ca. 0.2 eV). This makes CI_plan_ the sole deactivation path in azodicarboxamide, independent of the environment. The reason for this mechanism-determining alteration of the S_1_ topology is that in azodicarboxamide, the ES planarization decreases notably the S_0_–S_1_ energy gap, an effect not present in azobenzene. Upon decay to the GS, which is concluded in 400 fs, the electrostatic repulsion between the lone pairs derives the system out of planarity, allowing the switch to conclude the *pedalo* cycle within less than a picosecond.

The change of the photodynamics due to the different embedding of the azo-moiety within a molecule (no carbonyl groups in azobenzene vs. two carbonyl groups in azodicarboxamide) highlights, on the one hand, the potential of molecular design to modulate the light-responsive properties. On the other, it emphasizes the need for atomistic simulations of the excited-state dynamics that goes beyond the static picture in order to sort out the relevant pathways. This is particularly relevant in the case of diazocarboxamide, where the static description based on exploring the minimum energy path(s) from the FC to the S_1_/S_0_ CI seam favors the decay through a region of the seam (CI_cis-trans_) characterized by the pronounced twisting of the CNNC central fragment connecting the *cis* and *trans* isomers of dicarboxamide. The dynamics simulations demonstrate that, while being energetically more favorable, the system does not follow this decay path as momentum conservation along CNN/NNC bending coordinates and accessibility of CI_plan_ drive the system away from the minimum energy path.

The proposed mechanism has been corroborated by transient IR spectroscopy simulations from first principles tracking the dynamics of the carbonyl stretching around 1800 cm^−1^, an observer mode which is not directly involved in the isomerization but whose instantaneous frequency is affected by the electronic structure of the states visited in the course of the internal conversion. Upon ES planarization the delocalization of the frontier orbitals over the entire dicarboxamide unit leads to a weakening of the carbonyl bonds, which decreases the stretching frequency. Thus, the approx. 50 cm^−1^ red shift observed experimentally for the carbonyl band and reproduced in our atomistic simulations in solution and in gas-phase is a clear signature of the planarization completed in the ES before the decay to the GS.

In conclusion, we consider the argument regarding the azodicarboxamide decay mechanism as settled. The ascertainment of the dominant role of volume-conserving *pedalo* motion both in gas and liquid phase opens the door for applications of this novel switch in confined environments.

## Figures and Tables

**Table 1:** Structural parameters obtained at several critical points along the S_0_ and S_1_ potential energy surfaces of the gas-phase and solvated chromophore. The S_0_ minimum was obtained at the MP2/6-31G* level, whereas the remaining points at the SS-CASPT2/SA-2-CASSCF(18,12) level.

	MIN S_0_		Min S_1_		CI_cis-trans_		CI_plan_	
Parameters	Gas	CHCl_3_	Gas	CHCl_3_	Gas	CHCl_3_	Gas	CHCl_3_
α(N-C-N-N)	106.4°	103.1°	175.8°	178.8°	−179°	170°	178°	176°
β(N-N-C-N)	−107.4°	−115°	178.1°	176.1°	179°	170°	179°	178°
γ(C-N-N-C)	179.9°	175.9°	−174°	177.7°	−112°	125°	−160°	176°
CNN	109.7°	110°	125.2°	125.8°	123°	130°	135°	138°
NNC	109.8°	109.7°	123.6°	122.5°	124°	124°	136°	133°

**Table 2:** QM/MM vertical excitation energies (in eV/nm) and oscillator strengths (o.s.), where either CASPT2(18,12)/&-31G* or CAM-B3LYP/6-31G* are used for the QM calculation. CASPT2 and TD-DFT calculations are performed on the respective MP2 and DFT GS minimum. Electronic transitions refer to orbitals shown in Figure 2.

	CASPT2(18,12)			CAM-B3LYP		
	Electronic Transition (Weight)	Energy (eV/nm)	o.s.	Electronic Transition (Weight)	Energy (eV/nm)	o.s.
S_1_	n_N_ → π*_N_ (0.85)	2.64/470	0.00	n_N_ → π*_N_ (0.66)	2.79/444	0.00
S_2_	n_O2_ → π*_N_ (0.47) π_3_ → π*_N_ (0.28)	4.48/277	0.02	π_3_ → π*_N_ (0.67)	4.74/262	0.00
S_3_	π_3_ → π*_N_ (0.33) π_4_ → π*_N_ (0.31)	4.54/273	0.01	π_4_ → π*_N_ (0.69)	4.75/261	0.01
S_4_	π_4_ → π*_N_ (0.51) π_3_ → π*_N_ (0.19)	4.54/273	0.01	n_O2_ → π*_N_ (0.68)	5.02/247	0.03

## Data Availability

The data that support the findings of this study are available from the corresponding authors upon reasonable request.

## Supplementary Materials

The following supporting information can be downloaded at: https://www.mdpi.com/article/10.3390/molecules28020816/s1. QM/MM energy profiles along *pedalo*-type motion and *trans-cis* coordinates, molecular frontier orbitals in S_1_ *Plateau*, energy values along the minimum energy paths, values of bending angles at all S_1_/S_0_ hopping geometries, 0K molecular dynamics simulations, time-resolved UV pump/IR probe spectrum in gas phase and coordinates of critical points. Figure S1: Molecular frontier orbitals $\pi_{N}$ and $\pi_{N}^{*}$ obtained at a geometry from the *S_1_ Plateau* (see Figure 2 from main text) with N_1_C_2_N_3_N_4_, N_3_N_4_C_5_N_6_ and C_2_N_3_N_4_C_5_ exhibiting values close to 180°, Figure S2: left) QM/MM energy profiles along the *pedalo*-type coordinate optimizing either S_1_ (top) or S_0_ (bottom) at (TD)-DFT/CAM-B3LYP (open squares) and MP2/SS-CASPT2(18,12) (filled squares) level of theory. Right) QM/MM energy profiles along the *trans-cis* coordinate optimizing either S_1_ (top) or S_0_ (bottom) at (TD)-DFT/CAM-B3LYP (open squares) and MP2/SS-CASPT2(18,12) (filled squares), Table S1: Energy values of critical points along the minimum energy paths of the *pedalo*-type and *trans-cis* coordinates calculated at CASPT2(18,12) level, Figure S3: Values of the bending angles C_2_N_3_N_4_ ($\varphi_{CNN}$) and N_3_N_4_C_5_ ($\phi_{NNC}$) at all S_1_/S_0_ hopping geometries along the dynamics, Figure S4: 0K trajectories started from S_1_ in gas-phase (left) and solvent (right) until the hopping point with S_0_: 679 fs and 888 fs, respectively, Figure S5: Simulated Transient UV pump/IR probe obtained in gas phase at different delay times.
