# Supplementary material for: Modus Operandi of a Pedalo-Type Molecular Switch: Insight from Dynamics and Theoretical Spectroscopy
Source: Molecules. 2023 Jan 13;28(2):816. doi: 10.3390/molecules28020816 (PMC9863296; doi:10.3390/molecules28020816)
Supplement: Supplementary file 1 [file molecules-28-00816-s001.zip › molecules-2129496-supplementary.pdf]

Supporting Information

Modus Operandi of a Pedalo-Type Molecular Switch: Insight from Dynamics and Theoretical Spectroscopy

Mario Taddei [1], Marco Garavelli [1,*], Saeed Amirjalayer [2], Irene Conti [1,*] and Artur Nenov [1,*]

[1] Dipartimento di Chimica Industriale, Università degli Studi di Bologna, 40136 Bologna, Italy

[2] Center for Nanotechnology, Center for Multiscale Theory and Computation, Physikalisches Institut, Westfälische Wilhelms-Universität Münster, 48149 Münster, Germany

* Correspondence: marco.garavelli@unibo.it (M.G.); irene.conti@unibo.it (I.C.); artur.nenov@unibo.it (A.N.)

Relaxed scans along the pedalo-type and *trans-cis* coordinates

We calculated energy profiles along the *pedalo*-type and *trans-cis* coordinates. In particular, the former is characterized by the simultaneous torsion around C_2-N_3 and N_4-C_5 bonds in which dihedrals $N_1C_2N_3N_4$ and $N_3N_4C_5N_6$ rotate in opposite direction. By fixing these dihedrals every 10° between 90° and 180° while optimizing the other structural parameters we obtained the profile of S_0 at the DFT/CAM-B3LYP and MP2 level (Figure S2, bottom left), as well as the profile of S_1 at the SS-CASPT2/SA-2-CASSCF(18,12) level (Figure S2, top left)[1]. With both methods, the ground state energy minimum provides a distorted structure where NCNN and NNCN dihedral angles have values of about 110°, while in the first excited state the geometry with both torsional angle close to the planarity is the energetically preferred one.

Concerning the *trans-cis* reaction coordinate, energy profiles are obtained by fixing the $C_2N_3N_4C_5$ dihedral every 10° between 180° and 90° while optimizing the other degrees of freedom. The S_0 profile was computed at the DFT/CAM-B3LYP and MP2 level (Figure S2, bottom right), while the S_1 profile at the TD-DFT/CAM-B3LYP and SS-CASPT2/SA-2-CASSCF(18,12) levels (Figure S2, top left). The scan demonstrate that in the ground state the conformation with $C_2N_3N_4C_5$ dihedral equal to 180° (*trans* configuration) is the energetically preferred one. In the optimization of the first excited state, the energy profile at the SS-CASPT2/SA-2-CASSCF(18,12) level (blue dots in Figure S2, top left) is quite flat, gradually decreasing (by overall 0.2 eV) toward a crossing with the ground state (orange dots in Figure S2, top left) around 110°. At the TDDFT level the crossing occurs earlier along the torsional path, already at 140°, accompanied by a small barrier of ca. 0.15 eV.

[1] For the first excited state TDDFT failed to converge toward the crossing region.

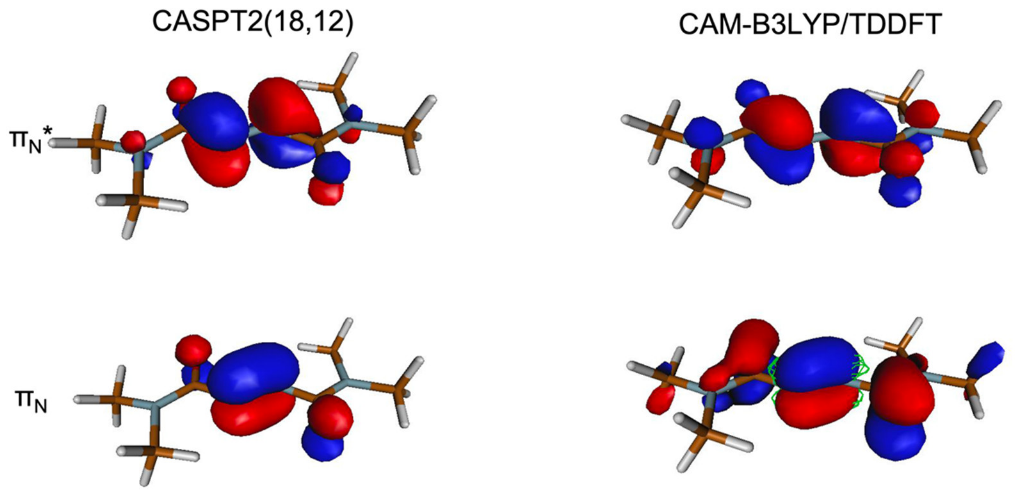

Figure S1. Molecular frontier orbitals π_N and π_N* obtained at a geometry from the *S_1 Plateau* (see Figure 2 from main text) with $N_1C_2N_3N_4$, $N_3N_4C_5N_6$ and $C_2N_3N_4C_5$ exhibiting values close to 180°.

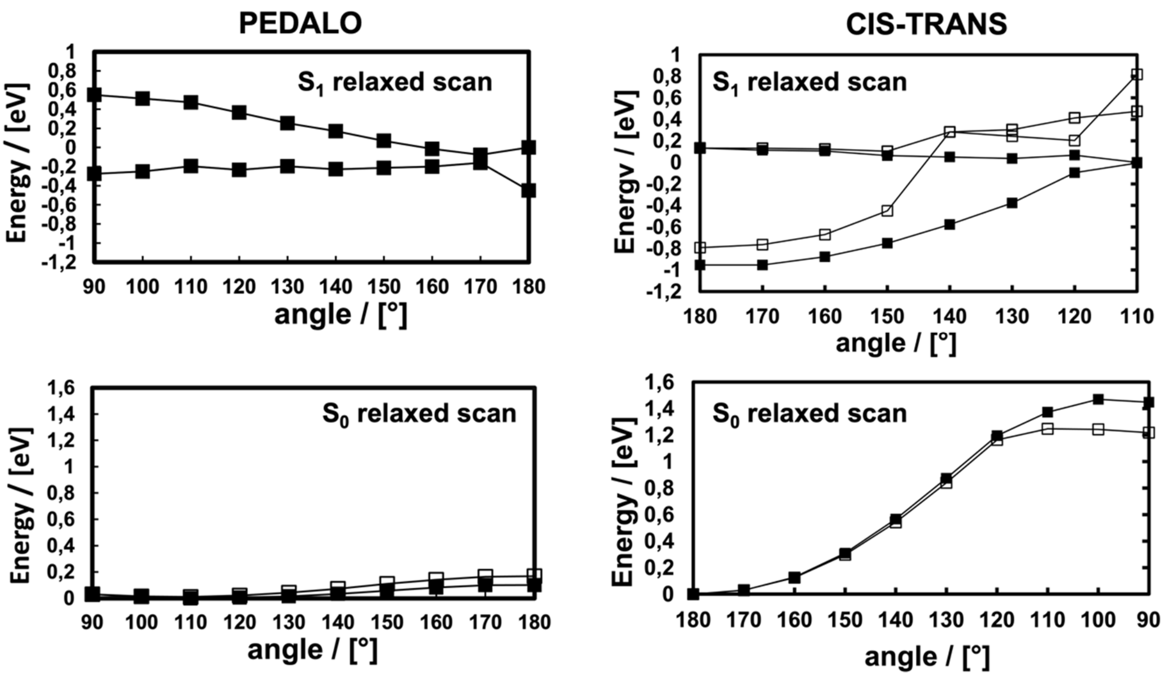

Figure S2. left) QM/MM energy profiles along the *pedalo*-type coordinate optimizing either S_1 (top) or S_0 (bottom) at (TD)-DFT/CAM-B3LYP (open squares) and MP2/SS-CASPT2(18,12) (filled squares) level of theory. right) QM/MM energy profiles along the *trans-cis* coordinate optimizing either S_1 (top) or S_0 (bottom) at (TD)-DFT/CAM-B3LYP (open squares) and SS-CASPT2(18,12) (filled squares).

Table S1. Energy values of critical points along the minimum energy paths of the *pedalo*-type and *trans-cis* coordinates calculated at CASPT2(18,12) level.

	FC	Plateau S_1	$CI_{cis\text{-}trans}$	CI_{plan}
S_0	0.00	0.86	1.73	2.00
S_1	2.81	1.84	1.73	2.00

Dynamics

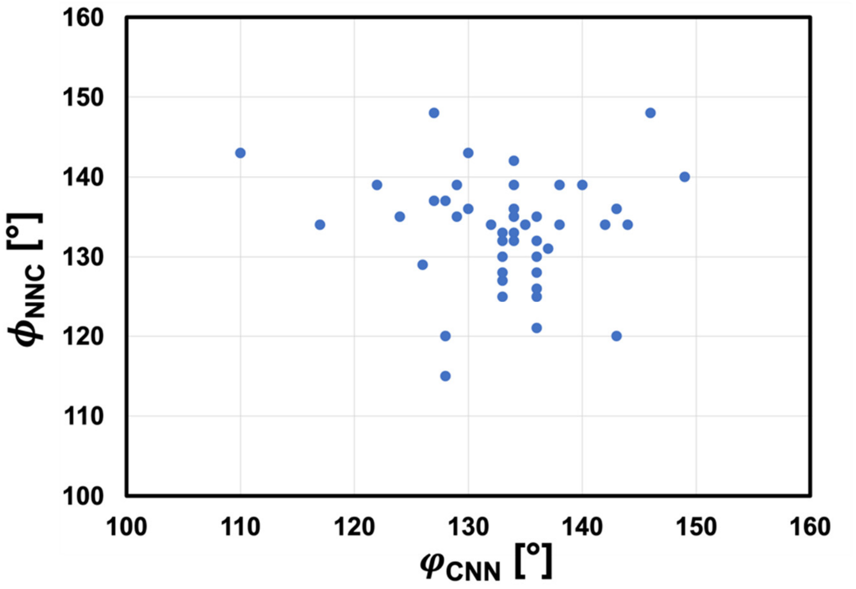

Figure S3. Values of the bending angles $C_2N_3N_4$ (φ_{CNN}) and $N_3N_4C_5$ (ϕ_{NNC}) at all S_1/S_0 hopping geometries along the dynamics.

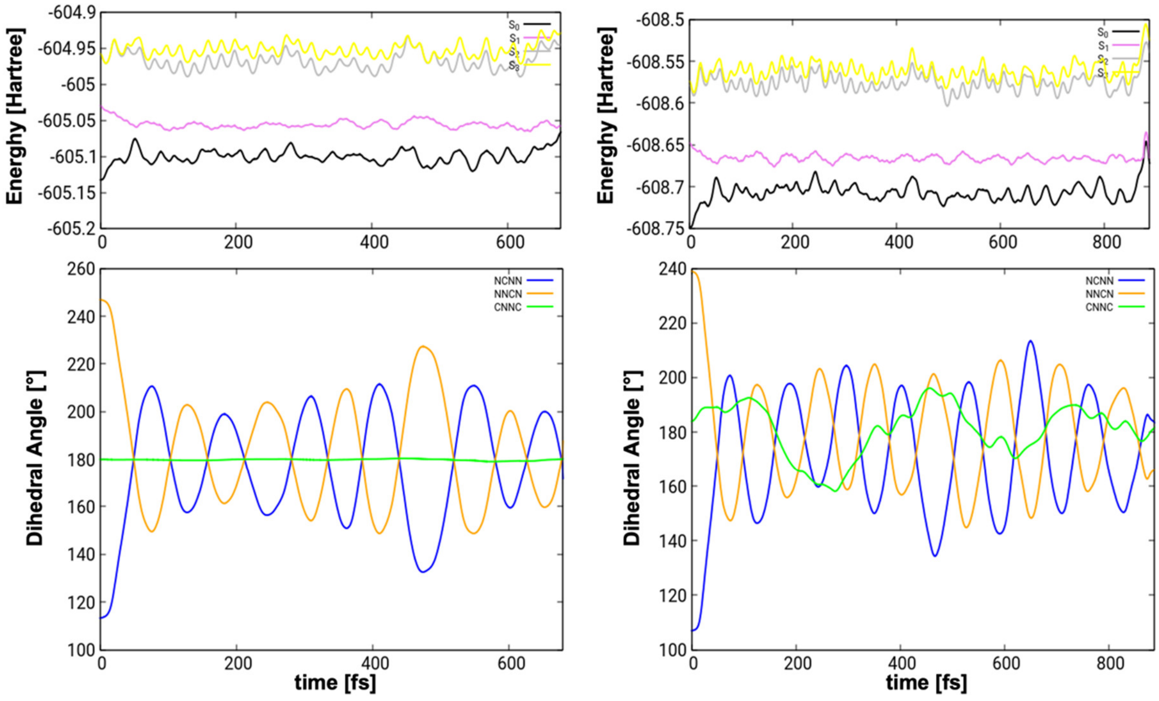

Figure S4. 0K trajectories started from S_1 in gas-phase (left) and solvent (right) until the hopping point with S_0: 679 fs and 888 fs, respectively.

Spectroscopy

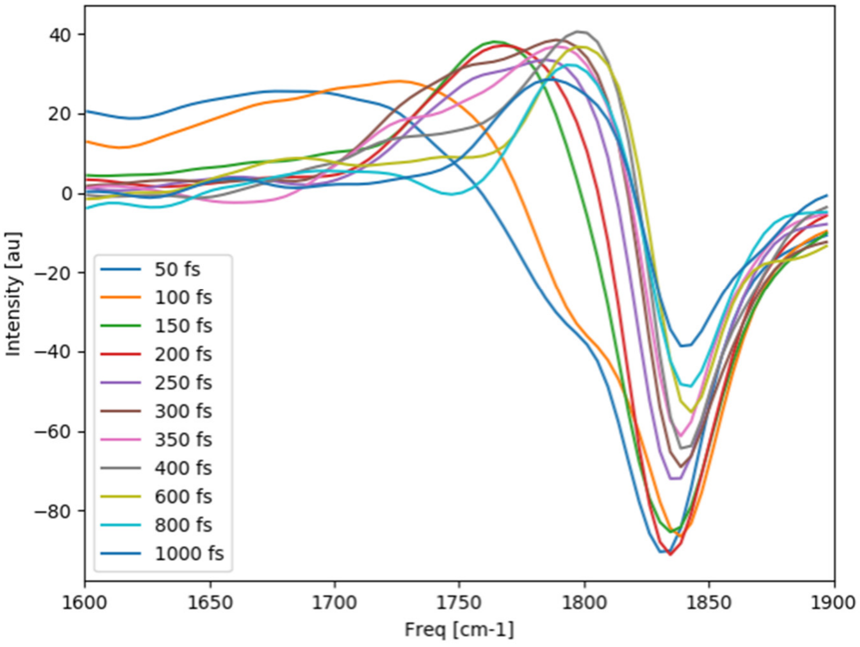

Figure S5. Simulated Transient UV pump / IR probe obtained in gas phase at different delay times.

Cartesian coordinates of all critical points (gas-phase) discussed in the main text

S_0 minimum (MP2)

O	23.063202	19.100840	15.635092
C	23.277499	19.708708	16.682717
N	22.150569	20.332940	17.372759
N	21.485107	19.448924	17.994710
C	20.357275	20.074431	18.682195
O	20.567327	20.667420	19.739207
N	24.472424	19.973087	17.253111
C	25.680238	19.516155	16.568412
C	26.366783	18.385502	17.323110
C	27.633221	18.573269	17.877170
C	28.250961	17.539820	18.580039
C	27.601731	16.316278	18.730909
C	26.339091	16.122987	18.174334
C	25.724114	17.156060	17.469906
C	24.615275	20.655158	18.542271
C	25.276158	22.018138	18.386913
C	26.583994	22.220363	18.828074
C	27.190130	23.466409	18.676548
C	26.487636	24.514466	18.084782
C	25.179343	24.317297	17.647101
C	24.574708	23.070849	17.799456
N	19.164959	19.828508	18.099148
C	19.025629	19.181649	16.791879
C	18.349612	17.821979	16.905734
C	17.046854	17.642379	16.440344
C	16.427021	16.399003	16.553797
C	17.110751	15.330861	17.131451
C	18.414081	15.505294	17.592874
C	19.032412	16.749089	17.478579
C	17.957488	20.297205	18.776321
C	17.346424	21.503187	18.074549
C	16.122515	21.388593	17.415061
C	15.570785	22.490972	16.764267
C	16.242197	23.712142	16.773145
C	17.463232	23.831406	17.434117
C	18.013200	22.728367	18.084687
H	25.385007	19.175640	15.576625
H	26.361384	20.365848	16.453248
H	23.636802	20.773262	19.003935
H	25.209741	20.019108	19.207116
H	18.442527	19.841757	16.140414
H	20.005934	19.065144	16.333793

H	18.235627	20.563064	19.795498
H	16.511110	18.472348	15.985260
H	15.410797	16.260505	16.189165
H	16.628382	14.358971	17.219282
H	18.950026	14.670642	18.041205
H	20.051382	16.881595	17.838618
H	17.237250	19.473821	18.818257
H	23.551934	22.920340	17.457737
H	24.628847	25.136267	17.187634
H	26.959323	25.488438	17.967215
H	28.210290	23.622650	19.022460
H	27.134346	21.405907	19.293835
H	28.143142	19.527068	17.761420
H	29.239494	17.687600	19.011081
H	28.082728	15.508953	19.280314
H	25.833025	15.166077	18.288907
H	24.736981	17.002655	17.036381
H	18.967611	22.824271	18.600313
H	17.987688	24.785208	17.443186
H	15.812133	24.573863	16.265755
H	14.615399	22.399732	16.250690
H	15.593838	20.438152	17.408074

S_0 minimum (DFT/CAM-B3LYP)

O	23.017651	19.060251	15.893107
C	23.302241	19.745865	16.856925
N	22.234251	20.427515	17.572156
N	21.539162	19.639232	18.222340
C	20.472081	20.320814	18.938959
O	20.758488	21.008097	19.901055
N	24.537032	20.045430	17.302432
C	25.680238	19.516155	16.568412
C	26.366783	18.385502	17.323110
C	27.633889	18.572673	17.875842
C	28.250961	17.539820	18.580039
C	27.601731	16.316278	18.730909
C	26.338064	16.123859	18.176137
C	25.722640	17.157320	17.472494
C	24.781057	20.815864	18.519716
C	25.460773	22.145797	18.221310
C	26.790108	22.355786	18.588391
C	27.411753	23.571051	18.306006
C	26.703361	24.580793	17.657381
C	25.373643	24.376443	17.293899
C	24.753536	23.160851	17.577166
N	19.236429	20.019163	18.497187
C	18.990229	19.246143	17.282022
C	18.311557	17.916629	17.584564
C	16.980596	17.706639	17.223433

C	16.359868	16.491825	17.509740
C	17.070795	15.482580	18.156376
C	18.402101	15.686984	18.513986
C	19.021293	16.902125	18.226800
C	18.094512	20.546314	19.234698
C	17.395532	21.667435	18.476231
C	16.118944	21.471962	17.948878
C	15.486510	22.495949	17.245567
C	16.129758	23.719642	17.069588
C	17.403376	23.920008	17.598537
C	18.034097	22.895273	18.301772
H	25.315035	19.151060	15.609138
H	26.388676	20.329475	16.376041
H	23.837554	20.999023	19.032154
H	25.400533	20.218891	19.199134
H	18.369158	19.841383	16.602541
H	19.932888	19.062287	16.768255
H	18.463673	20.920708	20.188830
H	16.423563	18.490155	16.714706
H	15.321569	16.329429	17.226232
H	16.587615	14.532921	18.379165
H	18.959111	14.897916	19.015967
H	20.062114	17.058401	18.505688
H	17.392565	19.729869	19.437607
H	23.713927	23.004694	17.293750
H	24.818564	25.165916	16.790419
H	27.187251	25.530802	17.437655
H	28.448780	23.733418	18.594141
H	27.345147	21.571979	19.098797
H	28.144740	19.525538	17.757936
H	29.239796	17.687356	19.010414
H	28.082831	15.508796	19.279948
H	25.831397	15.167401	18.291648
H	24.734644	17.004926	17.040518
H	19.029135	23.054575	18.714639
H	17.905687	24.876244	17.463758
H	15.636592	24.520338	16.521313
H	14.490064	22.341723	16.835568
H	15.612062	20.519650	18.086917

S_1 plateau (CASPT2(18,12)/SA-2-CASSCF)

O	23.183147	18.868841	16.000594
C	23.426704	19.585821	16.974627
N	22.414324	20.001778	17.875348
N	21.192869	19.698644	17.743935
C	20.139032	20.192100	18.546374
O	20.341422	20.903500	19.533664
N	24.646880	20.085684	17.326506
C	25.812827	19.588020	16.592841

C	26.466968	18.417077	17.317646
C	27.716646	18.568171	17.920182
C	28.307092	17.497992	18.591870
C	27.641853	16.273140	18.670132
C	26.397882	16.117762	18.058957
C	25.814714	17.185767	17.380967
C	24.872006	20.772873	18.597658
C	25.542769	22.125286	18.398960
C	26.858445	22.325339	18.816853
C	27.466403	23.568295	18.648684
C	26.757657	24.616115	18.064036
C	25.442792	24.420336	17.645757
C	24.836657	23.176471	17.814155
N	18.926515	19.750881	18.100650
C	18.761504	19.120034	16.791745
C	18.087329	17.757634	16.887591
C	16.790920	17.580550	16.403801
C	16.189316	16.324014	16.449449
C	16.885908	15.237996	16.975616
C	18.181833	15.409587	17.458569
C	18.781093	16.667726	17.413977
C	17.741384	20.277825	18.775217
C	17.187205	21.511500	18.070384
C	16.060507	21.406525	17.254641
C	15.573843	22.525575	16.581087
C	16.210666	23.756526	16.726185
C	17.330585	23.868790	17.547583
C	17.816190	22.748374	18.219286
H	25.479201	19.281264	15.601661
H	26.521306	20.413762	16.478821
H	23.909861	20.914955	19.091717
H	25.482991	20.138598	19.251498
H	18.180796	19.785769	16.141862
H	19.746454	19.004015	16.337420
H	18.023633	20.529125	19.796789
H	16.249802	18.420566	15.975454
H	15.180073	16.188096	16.064992
H	16.420540	14.254214	17.003070
H	18.728946	14.559635	17.862648
H	19.797748	16.795707	17.781181
H	16.985668	19.487988	18.811473
H	23.808170	23.027156	17.490561
H	24.888008	25.239239	17.191272
H	27.229668	25.588826	17.937299
H	28.492097	23.722944	18.978569
H	27.412023	21.512589	19.281464
H	28.232960	19.524546	17.868883
H	29.280778	17.617968	19.060991
H	28.095805	15.434752	19.193119
H	25.877634	15.163144	18.107916

H	24.844475	17.056109	16.903614
H	18.692830	22.841809	18.858204
H	17.827858	24.830130	17.664053
H	15.832436	24.631116	16.200107
H	14.697477	22.438697	15.941527
H	15.560488	20.448224	17.138978

S_1 plateau (TDDFT/CAM-B3LYP)

O	23.052166	19.050256	15.841648
C	23.293558	19.732768	16.824487
N	22.266622	20.322801	17.585310
N	21.045367	20.223978	17.381844
C	20.017832	20.752094	18.186066
O	20.257935	21.355033	19.219972
N	24.531444	20.036091	17.295818
C	25.680238	19.516155	16.568412
C	26.366783	18.385502	17.323110
C	27.633582	18.572847	17.876496
C	28.250961	17.539820	18.580039
C	27.601731	16.316278	18.730909
C	26.339383	16.122812	18.173574
C	25.724325	17.156039	17.469268
C	24.762313	20.788277	18.521490
C	25.369859	22.157888	18.245697
C	26.716608	22.399412	18.517554
C	27.271268	23.651363	18.256506
C	26.478042	24.666743	17.725530
C	25.130608	24.431279	17.459027
C	24.577889	23.178823	17.720561
N	18.780876	20.505934	17.680615
C	18.551490	19.895585	16.378369
C	17.965507	18.494300	16.497129
C	16.615528	18.268781	16.227442
C	16.081700	16.986359	16.344201
C	16.899111	15.924704	16.727442
C	18.249879	16.144756	16.990181
C	18.781701	17.427712	16.873352
C	17.631852	20.940331	18.461348
C	16.978741	22.186513	17.876001
C	15.734265	22.103319	17.251175
C	15.143974	23.243971	16.709264
C	15.796812	24.472302	16.793516
C	17.037738	24.560338	17.421456
C	17.626416	23.418859	17.962709
H	25.326075	19.154344	15.603931
H	26.387800	20.332927	16.385078
H	23.815070	20.909398	19.048340
H	25.422768	20.207909	19.176709
H	17.878620	20.538613	15.798593

H	19.496513	19.848999	15.836046
H	17.977605	21.148314	19.473292
H	15.975768	19.093600	15.922897
H	15.028142	16.812560	16.133217
H	16.484078	14.922402	16.816887
H	18.890677	15.315190	17.283390
H	19.838754	17.595583	17.072594
H	16.906163	20.120830	18.513029
H	23.523905	22.999062	17.516408
H	24.508995	25.225334	17.049332
H	26.909454	25.645380	17.522965
H	28.322200	23.837771	18.469834
H	27.337383	21.611126	18.936778
H	28.144120	19.525961	17.759822
H	29.239378	17.687733	19.011273
H	28.082732	15.508970	19.280308
H	25.833911	15.165480	18.287099
H	24.738201	17.002280	17.033658
H	18.595118	23.490803	18.455013
H	17.546922	25.519902	17.490996
H	15.336319	25.363934	16.371768
H	14.172759	23.176861	16.222400
H	15.219344	21.147695	17.186663

$CI_{cis\text{-}trans}$ (CASPT2(18,12)/SA-2-CASSCF)

O	23.094575	19.149602	16.178877
C	23.467187	19.912068	17.071132
N	22.538799	20.600920	17.916399
N	21.289297	20.513922	17.800322
C	20.458045	21.606682	17.389829
O	20.921772	22.719404	17.133565
N	24.755182	20.198518	17.395222
C	25.812827	19.588020	16.592841
C	26.466968	18.417077	17.317646
C	27.719485	18.566192	17.914612
C	28.307092	17.497992	18.591870
C	27.641853	16.273140	18.670132
C	26.393097	16.119696	18.071824
C	25.807899	17.189827	17.397323
C	25.133795	21.111972	18.467566
C	25.620502	22.452525	17.931201
C	26.979277	22.768361	17.959817
C	27.426562	23.991702	17.463045
C	26.514292	24.904699	16.937496
C	25.155995	24.594884	16.910852
C	24.710681	23.371708	17.407944
N	19.143138	21.261879	17.364630
C	18.667250	19.890675	17.523437
C	17.793414	19.734204	18.761484

C	16.438678	19.427500	18.631850
C	15.637809	19.295461	19.765166
C	16.191257	19.468522	21.032374
C	17.544969	19.771083	21.166228
C	18.344279	19.902246	20.032009
C	18.175157	22.289435	16.988580
C	17.709853	22.138382	15.545293
C	16.425849	21.669451	15.266213
C	16.003281	21.524765	13.945698
C	16.864976	21.850313	12.899929
C	18.147172	22.322084	13.174629
C	18.567577	22.466962	14.495529
H	25.368714	19.244810	15.659457
H	26.558724	20.352078	16.353464
H	24.274742	21.264915	19.120155
H	25.917045	20.633153	19.065309
H	18.106700	19.607964	16.624813
H	19.527498	19.225621	17.592016
H	18.649131	23.260268	17.125690
H	16.003207	19.289301	17.644789
H	14.581099	19.055769	19.661505
H	15.566841	19.365560	21.918051
H	17.978264	19.905102	22.155711
H	19.401011	20.139647	20.139506
H	17.321414	22.224902	17.670735
H	23.647642	23.138577	17.384213
H	24.440272	25.306455	16.502679
H	26.861422	25.860397	16.549065
H	28.487111	24.235268	17.485970
H	27.694922	22.059874	18.370172
H	28.241127	19.518285	17.853912
H	29.283734	17.617526	19.057491
H	28.099259	15.437715	19.197345
H	25.873919	15.164852	18.131448
H	24.830664	17.067033	16.933584
H	19.570048	22.835580	14.706613
H	18.821099	22.577044	12.358734
H	16.536694	21.737541	11.868252
H	15.001010	21.159042	13.730349
H	15.749858	21.416387	16.079654

CI_{plan} (CASPT2(18,12)/SA-2-CASSCF)

O	23.033614	19.210868	16.166259
C	23.452948	19.956769	17.056025
N	22.569820	20.651617	17.877939
N	21.317686	20.774150	17.963152
C	20.459457	21.712840	18.522771
O	20.914443	22.672562	19.153835
N	24.752585	20.200036	17.385799

C	25.812827	19.588020	16.592841
C	26.466968	18.417077	17.317646
C	27.726123	18.561546	17.901875
C	28.307092	17.497992	18.591870
C	27.641853	16.273140	18.670132
C	26.385018	16.125251	18.086676
C	25.796611	17.197785	17.418531
C	25.116743	21.044631	18.515358
C	25.593936	22.424523	18.077932
C	26.958324	22.692373	17.962557
C	27.395290	23.953968	17.561911
C	26.467256	24.954256	17.279396
C	25.103805	24.693578	17.400421
C	24.668586	23.431491	17.800541
N	19.145193	21.417130	18.318127
C	18.720335	20.290311	17.500557
C	18.089093	19.184349	18.336913
C	16.705535	19.005503	18.337083
C	16.128399	17.996112	19.105948
C	16.935202	17.160452	19.875776
C	18.317991	17.332897	19.874995
C	18.893317	18.342584	19.105518
C	18.138937	22.304845	18.889305
C	17.512673	23.209365	17.835152
C	16.202237	22.992247	17.408759
C	15.634363	23.816725	16.438665
C	16.376271	24.862964	15.893630
C	17.684144	25.085819	16.320000
C	18.250122	24.260554	17.290180
H	25.385349	19.250238	15.649307
H	26.557396	20.359036	16.368998
H	24.248992	21.144365	19.173558
H	25.898149	20.537706	19.090951
H	18.011286	20.648345	16.746248
H	19.587974	19.898316	16.961829
H	18.625042	22.908661	19.654900
H	16.072666	19.653068	17.734960
H	15.048661	17.858648	19.104274
H	16.485893	16.371425	20.476143
H	18.949133	16.679254	20.474328
H	19.973801	18.472975	19.107520
H	17.369163	21.696294	19.375281
H	23.602004	23.237323	17.898177
H	24.377781	25.475416	17.184862
H	26.806819	25.940319	16.967612
H	28.460330	24.159371	17.471492
H	27.686176	21.916214	18.186078
H	28.253820	19.509552	17.830985
H	29.286732	17.615348	19.051476
H	28.099494	15.437077	19.195849

H	25.861168	15.173541	18.154278
H	24.813577	17.079354	16.965700
H	19.271720	24.437504	17.622514
H	18.263763	25.904117	15.896731
H	15.933804	25.507840	15.136523
H	14.611723	23.645400	16.107677
H	15.618878	22.178989	17.833732
